# Decolonization of Human Anterior Nares of Staphylococcus aureus with Use of a Glycerol Monolaurate Nonaqueous Gel

**DOI:** 10.1128/mSphere.00552-20

**Published:** 2020-07-29

**Authors:** Patrick M. Schlievert, Marnie L. Peterson

**Affiliations:** a Department of Microbiology and Immunology, University of Iowa Carver College of Medicine, Iowa City, Iowa; b Hennepin Life Sciences, Minneapolis, Minnesota, USA; University of Nebraska Medical Center

**Keywords:** *Staphylococcus aureus*, coagulase-negative staphylococci, decolonization, glycerol monolaurate, nose

## Abstract

Staphylococcus aureus is a highly significant infection problem in health care centers, particularly after surgery. It has been shown that nearly 80% of S. aureus infections following surgery are the same as those in the anterior nares of patients, suggesting that the anterior nares is the source of the infection strain. This has led to the use of mupirocin ointment being applied nasally to reduce infections; mupirocin resistance is being observed. This study was undertaken to determine whether gel composed of 5% glycerol monolaurate solubilized in a glycol-based, nonaqueous gel (5% GML gel) could be used as an alternative. In our study, 40 healthy human volunteers swabbed their anterior nares for 3 days with the 5% GML gel. Prior to swabbing and 8 to 12 h after swabbing, S. aureus and coagulase-negative staphylococcal CFU per milliliter were determined by plating the swabs on mannitol salt agar. Fourteen of the volunteers had S. aureus in their nares prior to 5% GML gel treatment, most persons with the organisms present in both nares; five had pure cultures of S. aureus. All participants without pure culture of S. aureus were cocolonized with S. aureus and coagulase-negative staphylococci. Five of the S. aureus strains produced the superantigens commonly associated with toxic shock syndrome, though none of the participants became ill. For both S. aureus and coagulase-negative staphylococci, the 5% GML gel treatment resulted in a 3-log-unit reduction in microorganisms. For S. aureus, the reduction persisted for 2 or 3 days.

**IMPORTANCE** In this microflora study, we show that a 5% glycerol monolaurate nonaqueous gel is safe for use in the anterior nares. The gel was effective in reducing Staphylococcus aureus nasally, a highly significant hospital-associated pathogen. The gel may be a useful alternative or additive to mupirocin ointment for nasal use prior to surgery, noting that 80% of hospital-associated S. aureus infections are due to the same organism found in the nose. This gel also kills all enveloped viruses tested and should be considered for studies to reduce infection and transmission of coronaviruses and influenza viruses.

## Abstract (precis)

In this microflora study, we show that a 5% glycerol monolaurate nonaqueous gel is safe for use in the anterior nares. The gel was effective in reducing Staphylococcus aureus nasally, a highly significant hospital-associated pathogen. The gel may be a useful alternative or additive to mupirocin ointment for nasal use prior to surgery, noting that 80% of hospital-associated S. aureus infections are due to the same organism found in the nose. This gel also kills all enveloped viruses tested and should be considered for studies to reduce infection and transmission of coronaviruses and influenza viruses.

## INTRODUCTION

Staphylococcus aureus bacteria are common commensal bacteria in the nose and other mucosal surfaces of humans (1–4). Estimates of colonization rates are from 30 to 40% depending on age and underlying conditions. As many as 70% of humans may be transiently colonized. For nearly 80% of patients being treated for hospital-associated infections, the infecting S. aureus bacteria are the same as those in the anterior nares (3). This has led to the use of agents, such as mupirocin, to be applied to the nose prior to surgery (5–7). As might be expected, there is the appearance of mupirocin-resistant S. aureus (8).

Glycerol monolaurate (GML) is generally recognized as a safe compound by the Food and Drug Administration (FDA) for oral consumption and for use in cosmetics. This molecule is broadly antimicrobial for Gram-positive bacteria, including both methicillin-resistant S. aureus (MRSA) and methicillin-susceptible S. aureus (MSSA) (9). At approximately 50-fold-lower concentrations than the minimum bactericidal and minimum inhibitory concentrations, which are essentially the same for GML, the compound inhibits production of exotoxins (9). In human use studies, GML has been added to tampons and was shown to be safe (10). GML-coated tampons have been marketed in Europe to reduce the incidence of menstrual toxic shock syndrome (OptiBalance).

In subsequent studies, GML was mixed with a nonaqueous glycol-based gel at 5% GML (11–14). This gel has been referred to as 5% GML gel. In studies with 5% GML gel, it has been shown to be safe vaginally in chronic-use studies in rhesus macaques (6-month study) (12) and women (3-month study; unpublished data). The gel also reduces the transmission vaginally of multiple-challenge, high-dose simian immunodeficiency virus (13, 14). The 5% GML gel is highly active at killing both Gram-positive and Gram-negative bacteria, except lactobacilli, bifidobacteria, and certain enterococci (9, 12, 15). Resistant bacteria contain an immunity gene to GML, where GML acts as a quorum-sensing growth stimulant (15, 16). The 5% GML gel also prevents biofilm formation and removes preformed biofilms (9). The mechanism of action of the gel depends on GML dissipation of the potential difference across bacterial plasma membranes, with accompanying synergy by the nonaqueous gel component (9). As shown in vaginal studies, the glycol-based gel spreads laterally quickly to coat the vagina and other parts of the genital tract (17, 18). Because of the myriad of potential targets of 5% GML gel to kill bacteria, resistance to antimicrobial effects is limited (9).

Staphylococcal superantigens are a large family of secreted toxins that cause massive T lymphocyte proliferation (19, 20). Three of these toxins, notably toxic shock syndrome toxin 1 (TSST-1) and staphylococcal enterotoxins B and C (SEB and SEC), are the major causes of TSS (21). TSST-1 is the exclusive cause of menstrual TSS, occurring with mucosal colonization of S. aureus (22). In recent studies, it has been shown that there has been a significant increase in stains producing the six-member enterotoxin gene cluster of superantigens, at least since 2008 (23, 24). These six superantigens, including SEG and SE-like I, M, N, O, and U, appear to be more important for S. aureus colonization than overt disease causation (25).

This study was undertaken with institutional review board (IRB) approval to test the ability of 5% GML gel to reduce S. aureus colonization of the anterior nares of 40 humans. We confirmed that 35% of healthy volunteers were colonized with S. aureus. Strains were identified by the ability to produce the major superantigens that cause TSS, although no participants developed TSS. As in our prior studies, the enterotoxin gene cluster of superantigens was commonly present in S. aureus isolates. Five percent GML gel reduced S. aureus colonization significantly. Its antimicrobial effect persisted for up to 3 days.

## RESULTS

Of greatest importance, when queried upon completion of the study, none of the participants reported any adverse events with use of the 5% GML gel. Of the 40 participants, 14 were positive for S. aureus (35%) in the pre-GML gel treatment. Twelve of 14 individuals had S. aureus isolated from both nares. Five persons had pure cultures of S. aureus in both nares. The remaining nine individuals had mixtures of both S. aureus and coagulase-negative staphylococci in both nares.

The S. aureus isolates were analyzed for the presence of superantigen genes by PCR. All (100%) of the isolates contained the genes for one or more superantigens. Two strains had the ability to produce TSST-1, and the other 12 had the ability to produce SE-like X. There were no strains that had the genes for both TSST-1 and SE-like X. These two superantigens are usually not produced by the same strains (26). The reason for the exclusion remains unknown. Another notable feature of the superantigen profile was that 9/14 isolates contained components of the enterotoxin gene cluster of six superantigens, including SEG, SE-like I, M, N, O, and U (25, 27). This is consistent with the increased presence of these six superantigens in strains isolated at least since 2008 (24). None of the strains were positive for the SEB gene, while three were positive for the SEC gene. This means that at least five of the strains contained superantigen genes, where the superantigens are produced in high enough concentration to cause TSS (21). None of the individuals developed any sign of disease.

**FIG 1:**
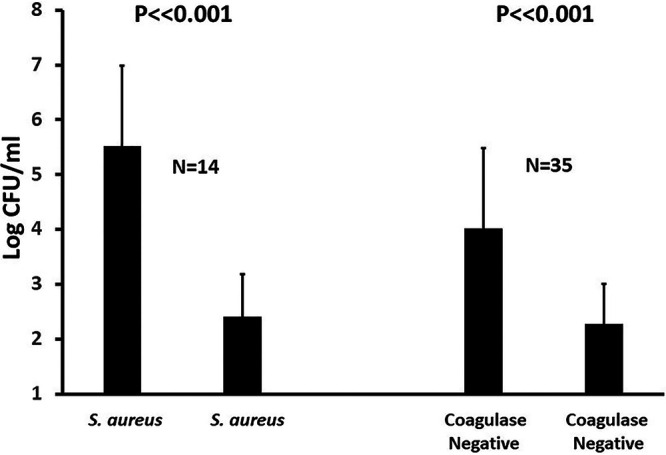
Effect of 5% GML nonaqueous gel on CFU of S. aureus and coagulase-negative staphylococci. The CFU/ml were log transformed prior to statistical analysis. Bars show mean CFU/ml before GML gel treatment (left bar) and after GML gel treatment (right bar). Data show mean CFU/ml plus standard deviation (SD) (error bar). *P* values were determined by Student’s *t* test.

The pre-GML gel and post-GML gel CFU per milliliter (CFU/ml) values were determined on the 14 individuals for both S. aureus and coagulase-negative staphylococci (Fig. 1); the data from both nares were included in the analysis, essentially giving 28 data points. As seen in Fig. 1, there were more than 10^5^ CFU/ml of S. aureus on average pre-GML gel treatment (log CFU/ml approximately 5.5). In contrast, after GML gel treatment for 3 days, the S. aureus counts fell to just over 10^2^/ml (log CFU/ml was approximately 2.2). Thus, there was a 3-log-unit reduction in S. aureus CFU/ml. Eight of the 14 participants (60%) had no detectable S. aureus in the nares after GML gel treatment.

GML gel also significantly reduced coagulase-negative staphylococci as present in the anterior nares (Fig. 1). Except for five individuals, where S. aureus was present in pure culture, all nine other persons had coagulase-negative staphylococci in the anterior nares with S. aureus. Additionally, all 26 individuals who did not have cultured S. aureus were positive for coagulase-negative staphylococci. Thus, 35 of the 40 participants had coagulase-negative staphylococci in their anterior nares before GML gel treatment.

**FIG 2:**
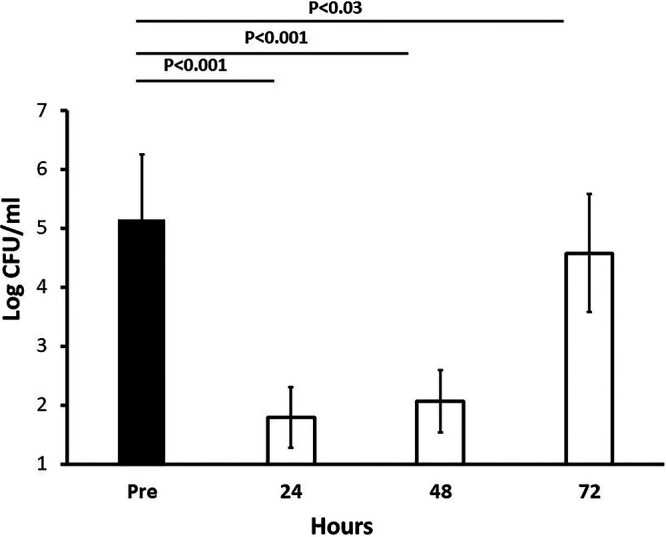
Persistence of 5% GML gel intranasal inhibition of S. aureus. Log-transformed CFU/ml from three individuals are shown. Values are means ± SD. The solid black bar shows the value pre-GML gel treatment. The white bars show the values at the indicated time (in hours) after GML gel treatment. *P* values were determined by Student’s paired *t* test.

In three individuals (six total data points at each time point), the persistence of reduction in S. aureus CFU/ml was evaluated (Fig. 2). The reduction in S. aureus CFU/ml persisted for 2 days before regrowth commenced as seen on day 3 after GML gel application.

## DISCUSSION

S. aureus causes more than 500,000 hospital-associated infections yearly in the United States. It has been shown that as much as 80% of the time, the S. aureus in the hospital-associated infection are the same as in the anterior nares (3). The data suggest that the anterior nares is the reservoir for the majority of hospital-associated S. aureus infections (2, 3).

The above observations have led to mupirocin in ointment form to be added to the anterior nares prior to surgery to reduce infections (5–7). Despite this, there remain a large number of infections. With these data in mind, the ability of 5% GML in a nonaqueous gel to reduce nasal S. aureus was evaluated in 40 humans.

The data showed that approximately 35% of adult humans had nasal S. aureus, and the percentage of persons positive is consistent with data from many other studies. The data also show that, when S. aureus was present, they were generally but not always present in both nares. In this study, 12.5% of healthy adults had S. aureus bacteria with the capability of producing large amounts of superantigens present, and thus, under the right conditions to cause TSS. For example, we described postinfluenza TSS in 1987 where 8/9 children succumbed to postinfluenza TSS, with 100% succumbing when TSST-1 was present (28); the other TSS isolate produced SEB. Additionally, TSST-1 is exclusively the cause of menstrual, vaginal TSS (22). Nine of 14 isolates contained components of the enterotoxin gene cluster of six superantigens. These six superantigens appear to be common in isolates, at least since 2008 (24). They appear to be more like colonization factors, as opposed to causing TSS (23, 25).

The current study is most significant because it shows that the 5% GML gel can be used to reduce S. aureus in the anterior nares significantly, and the effect lasts for at least 48 h posttreatment. There were no adverse events reported by any study participant. A prior study with rats, colonized nasally with S. aureus, obtained similar findings (29). The current data are significant for at least three reasons. (i) GML is generally recognized as safe by the FDA as a food and cosmetic additive. It is found in human breast milk at concentrations of about 3,000 μg/ml (30). Some underserved countries have used human breast milk to treat atopic dermatitis where S. aureus is commonly present (31). The gel component of the current mixture is nonaqueous, but the gel is already an approved class II medical device by FDA for human mucosal use. (ii) The GML gel as formulated has the ability to spread laterally to other parts of the nose. Although not tested in this study, K-Y warming gel, related to the gel used in this study, was shown in women to spread laterally after vaginal application to coat the genital tract (17, 18). Thus, if the movement of GML gel in the nares functions similarly, it would be expected to provide extensive coverage of the nose. (iii) The 5% GML gel is potently virucidal for all tested enveloped viruses, including influenza viruses and coronaviruses (13, 14, 32, 33). This makes 5% GML gel a possible preventative for viral transmission and nasal carriage. Subsequent studies will need to assess this *in vivo* in humans. However, in other studies, we have shown >90% effectiveness in preventing simian immunodeficiency virus transmission vaginally in rhesus macaques (13, 14).

For many years, mupirocin has been used topically, including nasally to reduce S. aureus colonization. For example, in one study of 68 health care workers, up to 6 months of treatment resulted in an 87% reduction in colonization rate (34). After only two treatments, there was a 58% reduction in colonization rate. Recolonization occurred with both the same and different S. aureus at an overall rate of 67% by 6 months. There were no reports of mupirocin resistance. However, in New Zealand, there was a steady increase in mupirocin resistance across the 1990s, reaching 28% (35). The overall rate of mupirocin resistance is variable, ranging typically from 3 to 4% up to 50%, depending on the study and health care setting (8). In the current study, after treatment with 5% GML gel, there was a 60% complete reduction in nasal colonization after 3 days treatment, comparable to mupirocin. We did not assess the long-term recolonization rate, but we did show that the S. aureus suppression lasted for 2 or 3 days. One advantage to use of 5% GML gel is the lack of S. aureus resistance to GML, even after 1 year of weekly passage on laboratory media at twofold below the MIC/minimum bactericidal concentration (9).

Overall, these studies suggest that 5% GML gel may be effective in reducing S. aureus hospital-associated infections. Because it has so many bacterial targets for inhibition, there is little chance of resistance developing. This is unlike mupirocin where resistant strains are arising. It may be possible to mix both 5% GML gel and mupirocin to increase effectiveness, while at the same time reducing resistance to mupirocin.

## MATERIALS AND METHODS

This study was a microflora study focused on S. aureus and coagulase-negative staphylococci. The study was performed under University of Minnesota IRB number 1103M97296 (nasal decolonization with glycerol monolaurate). The study was performed in the spring of 2011, and all participants were enrolled over a 2-week time period. There were 40 healthy volunteers, aged 18 to 64 years old, who completed the study, and 100% of enrollees completed the study. Each participant had their nares swabbed with prewetted saline (0.15 M NaCl) up to the nasal bones. The swabs were rotated three to five times during swabbing. Based on prior observation, it was assumed that each swab contained 0.1 ml of saline. The swabs were plated, with dilutions made, onto mannitol salt agar to select for staphylococci. Bacterial colonies that grew as bright yellow were then tested using catalase and slide coagulase tests to confirm S. aureus. Colonies that were red were considered coagulase-negative staphylococci.

The participants were next comparably swabbed with GML gel, for 3 days, approximately 12 h apart (twice per day). Finally, the participants returned to the laboratory 8 to 12 h after the last application to assess S. aureus CFU/ml by an additional swab. Three individuals were evaluated for an additional 24, 48, and 72 h after the final treatment for nasal S. aureus with no additional GML gel swabbed into the nares.

The isolated S. aureus strains were tested by PCR for the presence of superantigen genes (36). Superantigens have been shown in studies to be required for colonization and ability to cause human diseases (21, 25, 37). We did not assess the percentage of methicillin-resistant S. aureus.

Data were analyzed by Student’s paired *t* test by comparing log CFU/ml of S. aureus and coagulase-negative staphylococci in the pre-GML gel swabs compared to CFU/ml in the post-GML swabs.
